# Non-coding *NOTCH1* mutations in chronic lymphocytic leukemia; their clinical impact in the UK CLL4 trial

**DOI:** 10.1038/leu.2016.298

**Published:** 2016-11-11

**Authors:** M Larrayoz, M J J Rose-Zerilli, L Kadalayil, H Parker, S Blakemore, J Forster, Z Davis, A J Steele, A Collins, M Else, D Catovsky, D G Oscier, J C Strefford

**Affiliations:** 1 Caner Genomics, Academic Unit of Cancer Sciences, Faculty of Medicine, University of Southampton, Southampton, UK; 2 Genetic Epidemiology and Bioinformatics, Faculty of Medicine, University of Southampton, Southampton, UK; 3 Department of Molecular Pathology, Royal Bournemouth Hospital, Bournemouth, UK; 4 Division of Molecular Pathology, The Institute of Cancer Research, London, UK

In chronic lymphocytic leukemia (CLL), ‘coding' *NOTCH1* mutations were initially detected in exon 34 where they result in truncation of the C-PEST regulatory protein sequence with consequent impaired degradation of the Notch1 intracellular domain (NCID), constitutive activation of Notch signalling and increased cell survival and resistance to apoptosis.^1, 2, 3^ Mutations occur in 6–10% of cases at diagnosis, with increasing prevalence in advanced disease stages, treatment-refractory disease and after transformation to Richter syndrome.^4, 5^ In diagnostic and clinical trial cohorts, patients with *NOTCH1* mutations exhibited reduced survival.^5, 6^ In 2015, Puente and colleagues identified recurrent ‘non-coding' mutations clustered to the 3′-UTR of *NOTCH1* in 2% (11/506) previously untreated patients with CLL or monoclonal B-cell lymphocytosis.^7^ The presence of these 3′-UTR mutations cause a novel splicing event, preferentially between a cryptic donor site located in the last exon and a newly created acceptor site in the 3′-UTR of exon 34, resulting in the removal of the PEST sequence and constitutive activation of downstream signaling.^7^ Patients with non-coding *NOTCH1* mutations had similar outcomes to those with coding mutations, with shorter time to first treatment and shorter overall survival than wild-type cases.^7, 8^

Given the highly variable natural history of CLL and the often-serendipitous date of initial diagnosis, we aimed to establish the clinical significance of non-coding *NOTCH1* mutations in DNA samples available from 489 patients at enrolment to the United Kingdom Leukemia Research Fund Chronic Lymphocytic Leukemia 4 (UK LRF CLL4) chemotherapy trial.^9^ *NOTCH1* 3′-UTR mutations were identified by High Resolution Melt (HRM) analysis in whole genome amplified DNA (F: TGCTCGTTCAACTTCCCTTC; R: CAAGCAAGTTCTGAGAGCCA) and confirmed by Sanger sequencing of genomic DNA (F: CCTAACAGGCAGGTGATGCT; R: ATCTGGCCCCAGGTAGAAAC) The results were combined with the data pertaining to coding *NOTCH1* mutations in the same patient cohort from our previous publication.^5^ Fifty-three patients with wild-type HRM traces were sequenced, and no additional non-coding mutations were identified. It was not possible to differentiate between clonal and subclonal *NOTCH1* mutations using our HRM/Sanger approach. We defined associations between the presence of *NOTCH1* coding and non-coding mutation and a comprehensive panel of clinical and biological features reported in previous CLL4 papers,^10, 11, 12, 13^ by univariate logistic regression. Kaplan–Meier, log-rank test and Cox regression analysis were used to assess the impact of *NOTCH1* status on survival using Stata, where overall (OS) and progression-free (PFS) survival were defined as time from randomization to death from any cause and to relapse needing treatment, progression or death from any cause at last follow-up, respectively.

In addition to exon 34 coding mutations observed in 47/489 (9.6%) CLL4 patients*, w*e detected an additional 11/489 (2.2%) patients harbouring the non-coding mutations 139390152A>G (*n*=7) and 139390145A>G (*n*=4; Figure 1a), both previously reported to result in aberrant *NOTCH1* splicing.^7^ Importantly, the non-coding variants were mutually exclusive to coding variants, constituting 19% of the total *NOTCH1* mutational burden of CLL4 cases, with 11.8% of the patients carrying either type of *NOTCH1* mutation. *NOTCH1* non-coding mutations were not identified in cases with mutations of *TP53, BIRC3, BRAF* (V660E)*, MYD88* (L265P)*, NFKBIE* and *RPS15* mutations, but did co-occur with *SF3B1* (*n*=2) and *ATM* (*n*=2) mutations (Figure 1b). Next, we evaluated the association between the *NOTCH1* mutations and the main clinico-biological characteristics in CLL (Supplementary Table S1). As expected, when all 58 mutations were considered together, *NOTCH1* mutations were significantly more prevalent in CLL4 cases with unmutated *IGHV* genes (OR: 2.9, 95% CI: 1.4–6.2, *P*=0.005), CD38 (OR: 4.5, 95% CI: 2.3–8.7, *P*<0.001) and ZAP70 positivity (OR: 3.1, 95% CI: 1.5–6.4, *P*=0.002), high expression of CLLU1 (OR: 2.33, 95% CI: 1.2–4.4, *P*=0.01), trisomy 12 (OR: 4.0, 95% CI: 2.2–7.4, *P*<0.001) and ⩾15 × 10^9^/l absolute pro-lymphocytes (OR: 3.12, 95% CI: 2.0–7.9, *P*<0.001). However, for non-coding mutations on its own only the association with Trisomy 12 remained significant (OR: 5.6, 95% CI: 1.6–18.8, *P*=0.006), in spite of the limited number of cases with these mutations. Of the 364 deaths in CLL4 patients with the *NOTCH1* data, 14 (4%) were due to Richter's syndrome (RS). With non-coding *NOTCH1* mutations included, 4 of 14 (29%) Richter's deaths occurred in patients with *NOTCH1* mutation, an association that was non-significant (*P*=0.062).

In our previous CLL4 study, we confirmed the independent prognostic significance of a number of biomarkers, including coding *NOTCH1* mutations.^5^ In our current study, we determined the impact of coding and non-coding mutations on overall response rate (ORR), OS and PFS. Coding and non-coding mutations, inspected together or separately, were not associated with ORR in any of the three treatment arms (data not shown). Considered separately, univariate Cox regression analysis showed that patients with *NOTCH1* non-coding or coding mutations exhibited a significantly shorter OS (median survival times: 43.2 and 54.8 months, respectively) than patients with wild-type *NOTCH1* (median: 74.6 months). Non-coding and coding *NOTCH1* mutations were also associated with reduced PFS (median survival times: 22.0 and 13.0 months respectively) compared with the wild-type *NOTCH1* (28 months, Figure 1c and d). In further support of their clinical importance, cases with non-coding *NOTCH1* mutations showed a two-fold increase in the risk of mortality when compared with wild type (HR: 2.15, 95% CI: 1.17–3.92, *P*=0.013) and an 80% increase in the risk of progression or death (HR: 1.78, 95% CI: 0.98–3.24, *P*=0.05). The impact of coding and non-coding *NOTCH1* mutations together on OS was sustained in a multivariable model where *NOTCH1* status was controlled for gender, age, stage, *IGHV* and *SF3B1* mutational status, 11q deletion, and *TP53* mutation/ deletion (adjusted HR: 1.5, 95% CI: 1.0–2.1, *P*=0.04, Table 1). On the contrary, the association between *NOTCH1* mutational status and PFS was not significant when adjusted for the other variables listed above (adjusted HR: 1.3, 95% CI: 0.9–1.9, *P*=0.108). Taken together, we show that *NOTCH1* status, based on the presence of either mutational type, is an independent risk factor for OS but not for PFS. The association between OS or PFS and the occurrence of non-coding mutations could not be estimated reliably in a multivariable analysis because of the small number of cases with such mutations in our series.

Finally, we attempted to quantify the improved discriminatory power of including non-coding *NOTCH1* mutations to coding mutations as a test to predict both the presence and absence of PFS and OS events at last follow-up using sensitivity-specificity analysis. The analysis was carried out on all 489 cases. *NOTCH1* coding mutations correctly predicted 46/454 PFS (sensitivity of 10.1%) and 43/393 (sensitivity of 10.9%) OS events (Supplementary Table S2A and S3A). As expected, the sensitivity for OS and PFS was higher when both mutational types were considered than when coding mutation alone was analysed: 13.7 versus 10.9% for OS and 12.6 versus 10.1% for PFS events (Supplementary Table S2A and S3A). This increase reflected the fact that all 11 patients with non-coding *NOTCH1* mutations exhibited an adverse OS and PFS event, resulting in 100% specificity for non-coding *NOTCH1* mutation as a test. Accuracy assesses the capability of a given biomarker to correctly predict both the presence and absence of a survival event. Coding *NOTCH1* mutations displayed 16.4 and 27.6% accuracy for correctly predicting the presence or absence of a PFS and OS, respectively. Accuracy was increased to 18.6 and 29.9% for PFS and OS, respectively, when non-coding mutations were included in this analysis. The likelihood ratio, LR+, which adjusts sensitivity for false positives and LR−, which adjusts specificity for false negatives are prevalence-independent and their ratio, LR+/LR− (diagnostic odds ratio), is an indicator of the predictive power of the biomarker. A biomarker with a higher LR+/LR− value is a better predictor of the disease outcomes. Consistent with the increased sensitivity and higher accuracy, we observe increased LR+/LR− ratios for both PFS (3.81 versus 4.88) and OS (2.43 versus 3.66) when both coding and non-coding mutations were considered together (Supplementary Table S2A and S3A). In addition, the positive predictive value (PPV), which is a measure of the proportion of true positives out of all the outcomes predicted by the biomarker, is higher when non-coding mutation was included in the test than when coding-mutation alone was used as the test biomarker (98.3 versus 97.9% for PFS and 93.1 versus 91,5% for OS, Supplementary Table S2B and S3B).

In summary, our data confirm the prognostic importance of non-coding *NOTCH1* mutations in patients requiring first-line treatment with chemotherapy as part of the UK CLL4 trial. Importantly, restricted analysis of exon 34 neglected to identify 19% of patients with pathogenic *NOTCH1* mutations in its 3′-UTR region. In addition, we show that the discriminatory power of *NOTCH1* mutation status to predict outcomes is improved with the inclusion of non-coding mutations. Taken together, our study supports the analysis of the 3′-UTR region of the *NOTCH1* gene to identify additional patients with reduced survival. Several recent studies have provided conflicting data on the clinical significance of clonal and subclonal *NOTCH1* mutations.^8, 14, 15^ Most recently, Nadeu and colleagues demonstrated that the clonal mutations predicted for short OS, while subclonal mutations predicted for short time to first treatment.^9^ It will be important to employ these same deep sequencing approaches to ascertain the clinical significance of subclonal *NOTCH1* mutations in the clinical trials setting. The UK CLL4 trial benefits from long-term clinical follow-up and the expansive-associated clinico-biological data but only assessed the utility of traditional chemotherapy. Therefore, it will be necessary to establish the impact of non-coding *NOTCH1* mutations in patients treated with chemo-immunotherapy, where they are likely to identify a significant number of additional patients destined to respond poorly to rituximab-containing treatment regimens.^6^ Mutant *NOTCH1* currently represents a therapeutic target in T-ALL, with several mechanistic approaches under clinical development, including γ-secretase and metalloproteinases inhibitors, antibodies directed against the extracellular domain of Notch1 and antagonists that act by directly targeting the Notch transactivation domain. Screening for non-coding *NOTCH1* mutations identifies additional CLL patients with Notch1 activation, offering motivation for clinical trials development. Assuming these approaches are ultimately approved for the treatment of CLL, it will be critical to identify all patients that will benefit from these treatments, as there will be important clinical and cost implications. These studies will help establish a stratified and individualized approach to clinical management, including the more accurate selection of patients for targeted therapy.

## Figures and Tables

**Figure 1:**
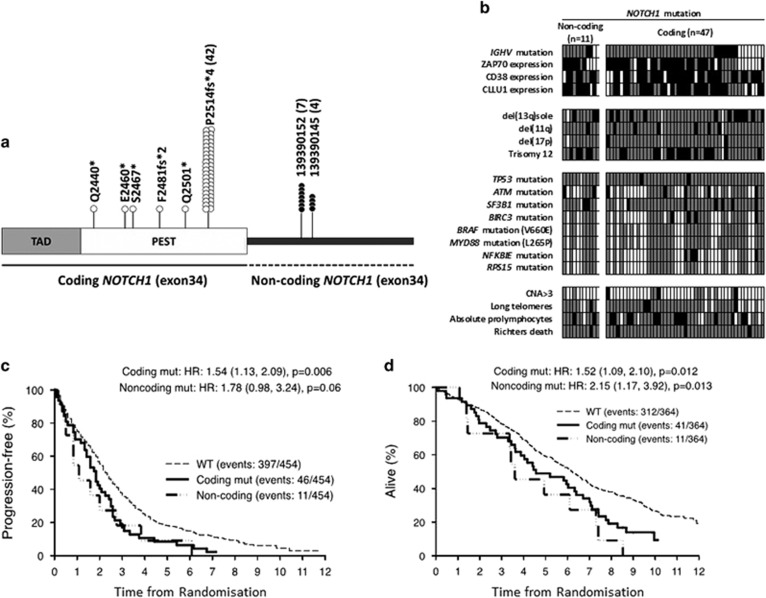
The genomic and clinical characteristics of *NOTCH1* non-coding and coding mutations in the LRF CLL4 trial. (**a**) The distribution of mutations in *NOTCH1*. The *NOTCH1* gene contains 34 exons and encodes a protein with a C-terminal TAD-PEST domain, which is a hotspot for mutation in CLL. Part of exon 34 and the 3′-UTR are magnified and the location of each mutation is shown; coding (white) and non-coding mutations (black) are indicated. Each dot represent a single mutation. (**b**) The mutual relationship between coding and non-coding *NOTCH1* mutations and other clinico-biological characteristics in CLL. Rows correspond to specific clinical and biological features and columns represent individual patients (only patients with a *NOTCH1* mutation are shown). Boxes colored black and grey show the presence or absence of a parameter. A white box denotes that no data were available. (**c**) and (**d**) Kaplan–Meir plots showing progression-free survival and overall survival, respectively.

**Table 1:** Univariate and multivariate Cox proportional hazard analysis of OS and PFS in CLL4 patients

*Variable*	*Overall survival*										*Progression-free survival*									
	*Univariate*							*Multivariate*			*Univariate*							*Multivariate*		
	*Total*	*Events*	*Median*	*95% CI*	*HR*	*95% CI*	*P*-value	*HR*	*95% CI*	*P*-value	*Total*	*Events*	*Median*	*95% CI*	*HR*	*95% CI*	*P*-value	*HR*	*95% CI*	*P*-value
NOTCH1																				
Wild type	431	312	74.6	67.8–81.5							431	394	27.6	24.9–30.4						
Mutated	58	52	53.4	35.9–70.9	1.6	1.2–2.2	0.001	1.5	1.0–2.1	0.04	58	57	19.3	15.0–23.5	1.6	1.2–2.1	0.001	1.3	0.9–1.9	0.108
																				
SF3B1																				
Wild type	364	250	79.1	71.8–86.3							364	326	26.5	23.1–29.9						
Mutated	73	66	54.3	47.3–61.4	1.7	1.3–2.2	<0.001	1.5	1.1–2.1	0.014	73	73	26.5	22.4–30.7	1.3	1.0–1.7	0.033	1.3	0.9–1.8	0.071
																				
Age																				
					1.1	1.0–1.1	<0.001	1.1	1.0–1.1	<0.001					1	0.9–1.1	0.663	0.9	0.9–1.0	0.387
																				
*Sex*																				
Male	366	281	70.1	61.4–78.9							366	341	25.0	21.9–28.0						
Female	129	86	79.6	66.5–93.0	0.8	0.6–1.0	0.056	0.8	0.6–1.1	0.121	129	115	29.4	25.5–33.3	0.8	0.7–1.0	0.055	0.9	0.7–1.1	0.338
																				
*Binet stag*e																				
A	112	76	80.6	63.4–97.7							112	104	27.2	23.8–30.7						
B/C	383	291	71.5	64.6–78.3	1.3	1.0–1.7	0.049	1.5	1.1–2.1	0.013	383	352	26.1	23.0–29.1	0.9	0.8–1.3	0.995	1.2	0.9–1.5	0.433
																				
*Del(11q)*																				
Undeleted	373	267	75	67.5–82.6							373	267	75	67.4–82.6						
Deleted	92	79	57.7	42.4–73.0	1.6	1.3–2.1	<0.001	1.4	1.1–1.9	0.023	92	79	57.7	42.4–73.0	1.5	1.2–1.9	0.001	1.7	1.3–2.2	<0.001
																				
IGHV *status*																				
Mutated	155	91	104.2	93.3–115.1							155	91	104.2	93.3–115.1						
Unmutated	255	216	60.6	52–8–68.4	2.2	1.7–2.8	<0.001	1.9	1.4–2.5	<0.001	255	216	60.6	52.8–68.4	1.9	1.6–2.4	<0.001	1.8	1.4–2.4	<0.001
																				
TP53 *status*																				
Normal	431	313	75.9	69.3–82.1							431	313	75.9	69.7–82.1						
Del/Mut	32	31	26.1	4.9–47.4	3.1	2.2–4.6	<0.001	2.5	1.5–4.1	<0.001	32	31	26.1	4.9–47.4	2.7	1.9–3.9	<0.001	2.2	1.3–3.5	0.002
																				
*Treatment arm*																				
Chl	238	178	76.8	70.1–83.4							238	178	76.8	70.1–83.4						
FDR/FC	257	189	68	57.9–78.1	1.1	0.9–1.3	0.426	0.9	0.8–1.3	0.854	257	189	68	57.9–78.1	0.6	0.5–0.7	<0.001	0.5	0.4–0.6	<0.001

Abbreviations: Chl, chlorambucil; FC, fludarabine plus cyclophosphamide; FDR: fludarabine.

OS multivariate, 342 cases with 252 events; 153 missing data. PFS multivariate, 342 cases with 315 events, 153 missing data.
