# Supplementary material for: Non-coding NOTCH1 mutations in chronic lymphocytic leukemia; their clinical impact in the UK CLL4 trial
Source: Leukemia. 2016 Nov 11;31(2):510–4. doi: 10.1038/leu.2016.298 (PMC5289571; doi:10.1038/leu.2016.298)
Supplement: Supplementary Tables [file leu2016298x1.docx]

**Supplementary Table1. Association between *NOTCH1* mutations and clinico-biological characteristics in CLL4 patients.**

| **Variable** | **OR** | **95% CI** | **P** | **N^1^** |
| --- | --- | --- | --- | --- |
| Age at diagnosis | 1.049 | 1.02-1.09 | 0.005 | 489 |
| Sex (male) | 0.64 | 0.32-1.27 | 0.198 | 489 |
| Binet stage (B and C) | 1.02 | 0.53-1.97 | 0.956 | 489 |
| CD38 positive^2^ | 4.48 | 2.30-8.72 | <0.0001 | 398 |
| ZAP70 positive^3^ | 3.11 | 1.52-6.37 | 0.002 | 370 |
| IGHVunmutated^4^ | 2.93 | 1.38-6.23 | 0.005 | 405 |
| *TP53* del/mut | 1.056 | 0.36-3.13 | 0.922 | 460 |
| del(11q) | 0.54 | 0.24-1.23 | 0.144 | 462 |
| del(13q) sole | 0.56 | 0.30-1.05 | 0.07 | 459 |
| Trisomy 12 | 4 | 2.16-7.38 | <0.0001 | 462 |
| Response to treatment^5^ | 0.98 | 0.67-1.44 | 0.933 | 462 |
| Treatment arm^6^ (CHL vs FD/FDR) | 0.63 | 0.43-0.92 | 0.018 | 489 |
| Higher CLLU1 expression^7^ | 2.33 | 1.23-4.43 | 0.01 | 398 |
| Long Telomere^8^ | 0.5 | 0.30-.85 | 0.011 | 345 |
| Absolute prolymphocyte range | 3.97 | 1.99-7.91 | <0.0001 | 380 |
| Richters death | 3.12 | 0.95-10.29 | 0.062 | 489 |

^1^ Shows the number of observations included in each logistic regression analysis.

^2^ CD38 positive ≥30%

^3^ ZAP70 positive≥10%

^4^ IGHV unmutated≥98%

^5^ Response: CR (complete response), nPR (nodular partial response) or PR (partial response) versus NR (non-response)

^6^ Treatment: CHL (Chlorambucil), FDR (fludarabine) or FD (fludarabine plus cyclophosphamide).

^7^ High CLLU1 expression≥40

^8^ Long telomere as defined by Strefford *et al* (2015), Leukemia 27(11):2196-9

**Supplementary Table 2A. Sensitivity and specificity analysis of *NOTCH1* mutation (n=489*) for progression-free survival**

| **NOTCH1 mutation** |  | **Sensitivity**  **(TPR^¶^)** |  | **Specificity**  **(TNR^¶^)** |  | **False Negative Rate**  **(FNR)** |  | **False Positive Rate**  **(FPR)** |  | **Accuracy** |  | **Likelihood Ratio**  **(LR^+^)^¶^** |  | **Likelihood Ratio**  **(LR^-^)^¶^** |  | **LR^+^/ LR^-^** |
| --- | --- | --- | --- | --- | --- | --- | --- | --- | --- | --- | --- | --- | --- | --- | --- | --- |
| Coding or non-coding mutation |  | 12.6%  (57/454) |  | 97.1%  (34/35) |  | 87.4%  (397/454) |  | 2.9%  (1/35) |  | 18.6%  (91/489) |  | 4.39 |  | 0.90 |  | 4.88 |
| Coding mutations |  | 10.1%  (46/454) |  | 97.1%  (34/35) |  | 89.9%  (408/454) |  | 2.9%  (1/35) |  | 16.4%  (80/489) |  | 3.54 |  | 0. 93 |  | 3.81 |
| Non-coding mutation |  | 2.4%  (11/454) |  | 100%  (11/11) |  | 97.6%  (443/454) |  | 0%  (0/35) |  | 9.4%  (46/489) |  | NE |  | 0.98 |  | NE |

*With data for *NOTCH1* status.

**^¶^**TPR=True Positive Rate, TNR=True Negative Rate, LR^+^=TPR/FPR; LR^-^=TNR/FNR

**Supplementary Table 2B. Positive and negative predictive values for *NOTCH1* mutations and progression-free survival**

| ***NOTCH1* mutation** |  | **Positive Predictive Value (PPV)** |  | **Negative Predictive Value (NPV)** |
| --- | --- | --- | --- | --- |
| Coding or non-coding mutation |  | 98.3%  (57/58) |  | 7.0%  (34/431) |
| Coding mutations |  | 97.9%  (46/47) |  | 7.7%  (34/442) |
| Non-coding mutation |  | 100%  (11/11) |  | 7.3%  (35/478) |

**Supplementary Table 3A. Sensitivity and specificity analysis of NOTCH1 mutations (n=489*) for overall survival**

| ***NOTCH1* mutation** |  | **Sensitivity**  **(TPR^¶^)** |  | **Specificity**  **(TNR^¶^)** |  | **False Negative Rate**  **(FNR)** |  | **False Positive Rate**  **(FPR)** |  | **Accuracy** |  | **Likelihood Ratio**  **(LR^+^)^¶^** |  | **Likelihood Ratio**  **(LR^-^)^¶^** |  | **LR^+^/ LR^-^** |
| --- | --- | --- | --- | --- | --- | --- | --- | --- | --- | --- | --- | --- | --- | --- | --- | --- |
| Coding or non-coding mutation |  | 13.7%  (54/393) |  | 95.8%  (92/96) |  | 86.3%  (339/393) |  | 4.2%  (4/96) |  | 29.9%  (146/489) |  | 3.29 |  | 0.90 |  | 3.66 |
| Coding mutations |  | 10.9%  (43/393) |  | 95.8%  (92/96) |  | 89.1%  (350/393) |  | 4.2%  (4/96) |  | 27.6%  (160/489) |  | 2.62 |  | 1. 08 |  | 2.43 |
| Non-coding mutation |  | 2.8%  (11/393) |  | 100%  (96/96) |  | 97.2%  (382/393) |  | 0%  (0/96) |  | 21.9%  (107/489) |  | NE |  | 0.97 |  | NE |

*With data for *NOTCH1* status.

**^¶^**TPR=True Positive Rate, TNR=True Negative Rate, LR^+^=TPR/FPR; LR^-^=TNR/FNR

**Supplementary Table 3B. Positive and negative predictive values for *NOTCH1* mutations and overall survival**

| ***NOTCH1* mutation** |  | **Positive Predictive Value (PPV)** |  | **Negative Predictive Value (NPV)** |
| --- | --- | --- | --- | --- |
| Coding or non-coding mutation |  | 93.1%  (54/58) |  | 27.6%  (119/431) |
| Coding mutations |  | 91.5%  (43/47) |  | 20.8%  (92/442) |
| Non-coding mutation |  | 100%  (11/11) |  | 20.1%  (96/478) |
